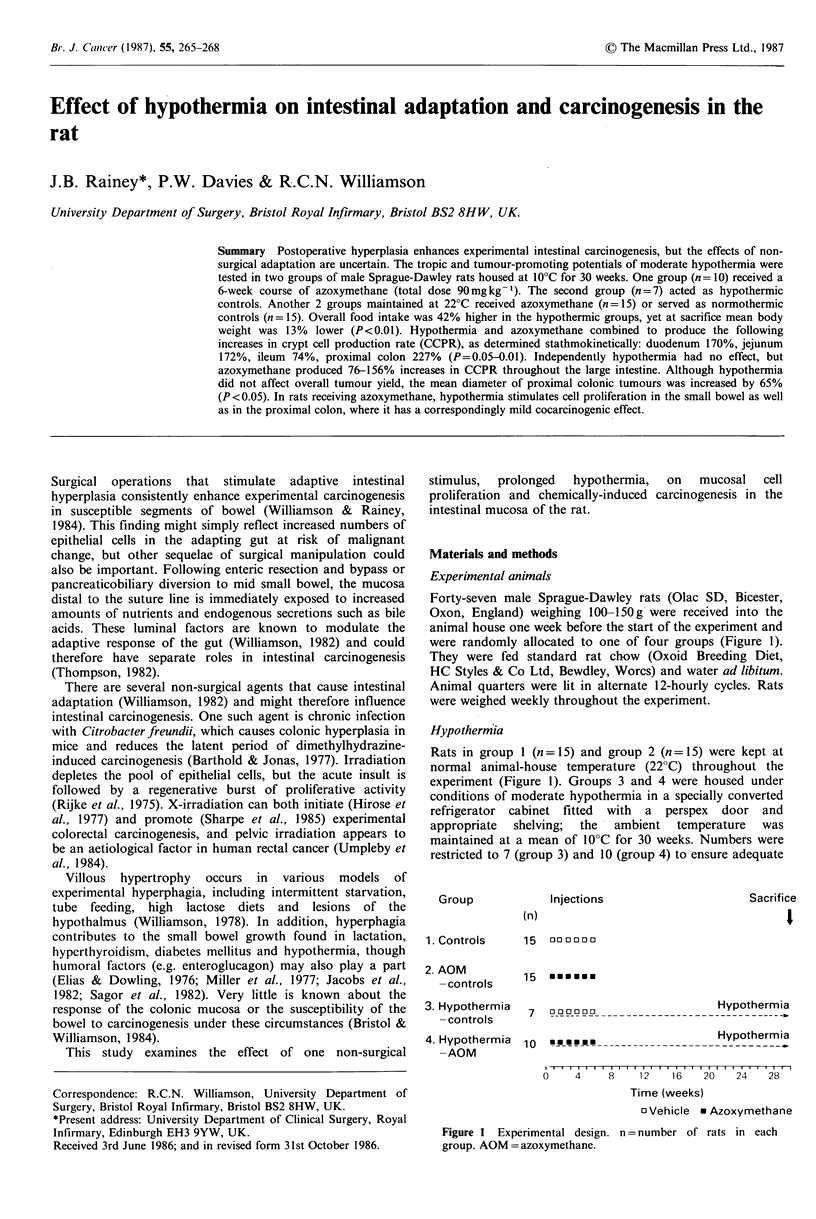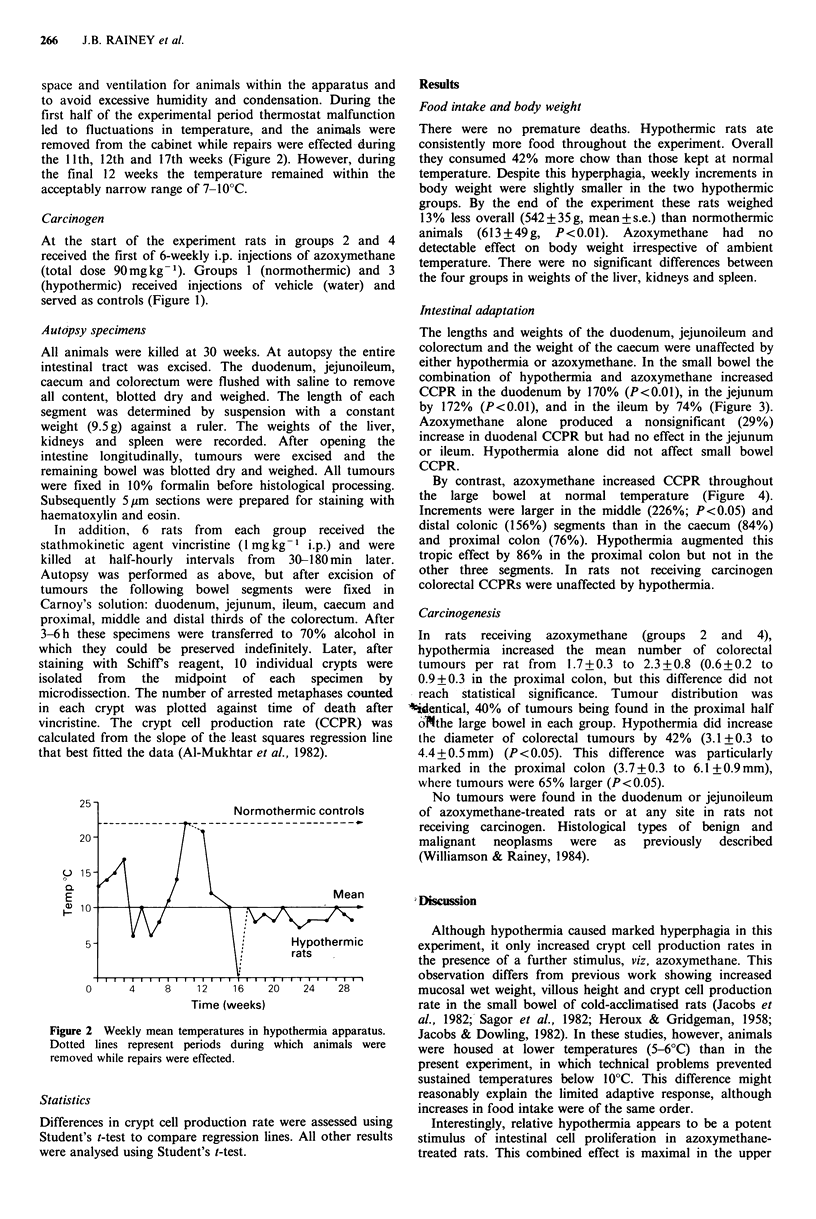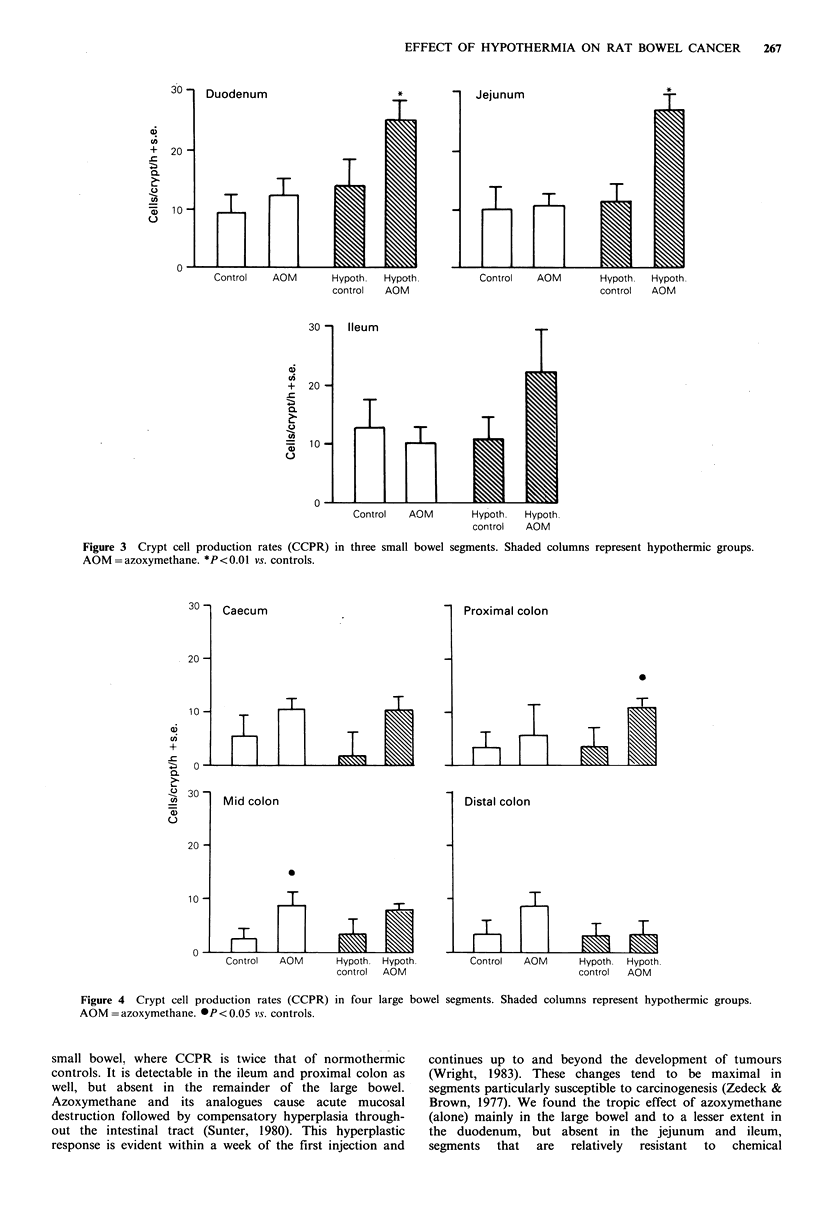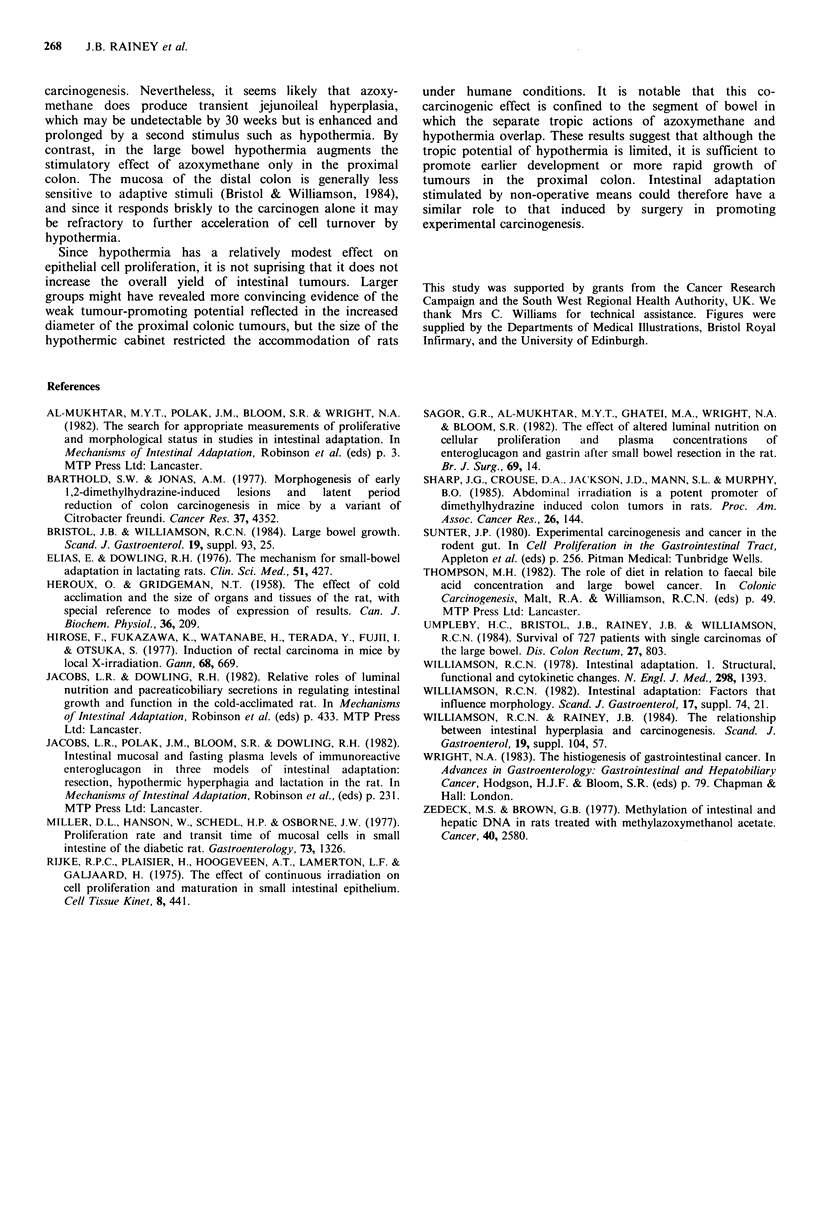# Effect of hypothermia on intestinal adaptation and carcinogenesis in the rat.

**DOI:** 10.1038/bjc.1987.51

**Published:** 1987-03

**Authors:** J. B. Rainey, P. W. Davies, R. C. Williamson

## Abstract

Postoperative hyperplasia enhances experimental intestinal carcinogenesis, but the effects of nonsurgical adaptation are uncertain. The tropic and tumour-promoting potentials of moderate hypothermia were tested in two groups of male Sprague-Dawley rats housed at 10 degrees C for 30 weeks. One group (n = 10) received a 6-week course of azoxymethane (total dose 90 mg kg-1). The second group (n = 7) acted as hypothermic controls. Another 2 groups maintained at 22 degrees C received azoxymethane (n = 15) or served as normothermic controls (n = 15). Overall food intake was 42% higher in the hypothermic groups, yet at sacrifice mean body weight was 13% lower (P less than 0.01). Hypothermia and azoxymethane combined to produce the following increases in crypt cell production rate (CCPR), as determined stathmokinetically: duodenum 170%, jejunum 172%, ileum 74%, proximal colon 227% (P = 0.05-0.01). Independently hypothermia had no effect, but azoxymethane produced 76-156% increases in CCPR throughout the large intestine. Although hypothermia did not affect overall tumour yield, the mean diameter of proximal colonic tumours was increased by 65% (P less than 0.05). In rats receiving azoxymethane, hypothermia stimulates cell proliferation in the small bowel as well as in the proximal colon, where it has a correspondingly mild cocarcinogenic effect.


### Effect of hypothermia on intestinal adaptation and carcinogenesis in the rat

J.B. Rainey*, P.W. Davies & R.C.N. Williamson

*University Department of Surgery, Bristol Royal Infirmary, Bristol BS2 8HW, UK.*

**Summary** Postoperative hyperplasia enhances experimental intestinal carcinogenesis, but the effects of non-surgical adaptation are uncertain. The tropic and tumour-promoting potentials of moderate hypothermia were tested in two groups of male Sprague-Dawley rats housed at 10°C for 30 weeks. One group ($n=10$) received a 6-week course of azoxymethane (total dose $90\,mg\,kg^{-1}$). The second group ($n=7$) acted as hypothermic controls. Another 2 groups maintained at 22°C received azoxymethane ($n=15$) or served as normothermic controls ($n=15$). Overall food intake was 42% higher in the hypothermic groups, yet at sacrifice mean body weight was 13% lower ($P<0.01$). Hypothermia and azoxymethane combined to produce the following increases in crypt cell production rate (CCPR), as determined stathmokinetically: duodenum 170%, jejunum 172%, ileum 74%, proximal colon 227% ($P=0.05$–$0.01$). Independently hypothermia had no effect, but azoxymethane produced 76–156% increases in CCPR throughout the large intestine. Although hypothermia did not affect overall tumour yield, the mean diameter of proximal colonic tumours was increased by 65% ($P<0.05$). In rats receiving azoxymethane, hypothermia stimulates cell proliferation in the small bowel as well as in the proximal colon, where it has a correspondingly mild cocarcinogenic effect.

Surgical operations that stimulate adaptive intestinal hyperplasia consistently enhance experimental carcinogenesis in susceptible segments of bowel (Williamson & Rainey, 1984). This finding might simply reflect increased numbers of epithelial cells in the adapting gut at risk of malignant change, but other sequelae of surgical manipulation could also be important. Following enteric resection and bypass or pancreaticobiliary diversion to mid small bowel, the mucosa distal to the suture line is immediately exposed to increased amounts of nutrients and endogenous secretions such as bile acids. These luminal factors are known to modulate the adaptive response of the gut (Williamson, 1982) and could therefore have separate roles in intestinal carcinogenesis (Thompson, 1982).

There are several non-surgical agents that cause intestinal adaptation (Williamson, 1982) and might therefore influence intestinal carcinogenesis. One such agent is chronic infection with *Citrobacter freundii*, which causes colonic hyperplasia in mice and reduces the latent period of dimethylhydrazine-induced carcinogenesis (Barthold & Jonas, 1977). Irradiation depletes the pool of epithelial cells, but the acute insult is followed by a regenerative burst of proliferative activity (Rijke *et al.*, 1975). X-irradiation can both initiate (Hirose *et al.*, 1977) and promote (Sharpe *et al.*, 1985) experimental colorectal carcinogenesis, and pelvic irradiation appears to be an aetiological factor in human rectal cancer (Umpleby *et al.*, 1984).

Villous hypertrophy occurs in various models of experimental hyperphagia, including intermittent starvation, tube feeding, high lactose diets and lesions of the hypothalmus (Williamson, 1978). In addition, hyperphagia contributes to the small bowel growth found in lactation, hyperthyroidism, diabetes mellitus and hypothermia, though humoral factors (e.g. enteroglucagon) may also play a part (Elias & Dowling, 1976; Miller *et al.*, 1977; Jacobs *et al.*, 1982; Sagor *et al.*, 1982). Very little is known about the response of the colonic mucosa or the susceptibility of the bowel to carcinogenesis under these circumstances (Bristol & Williamson, 1984).

This study examines the effect of one non-surgical stimulus, prolonged hypothermia, on mucosal cell proliferation and chemically-induced carcinogenesis in the intestinal mucosa of the rat.

### Materials and methods

### *Experimental animals*

Forty-seven male Sprague-Dawley rats (Olac SD, Bicester, Oxon, England) weighing 100–150 g were received into the animal house one week before the start of the experiment and were randomly allocated to one of four groups (Figure 1). They were fed standard rat chow (Oxoid Breeding Diet, HC Styles & Co Ltd, Bewdley, Worcs) and water *ad libitum*. Animal quarters were lit in alternate 12-hourly cycles. Rats were weighed weekly throughout the experiment.

### *Hypothermia*

Rats in group 1 ($n=15$) and group 2 ($n=15$) were kept at normal animal-house temperature (22°C) throughout the experiment (Figure 1). Groups 3 and 4 were housed under conditions of moderate hypothermia in a specially converted refrigerator cabinet fitted with a perspex door and appropriate shelving; the ambient temperature was maintained at a mean of 10°C for 30 weeks. Numbers were restricted to 7 (group 3) and 10 (group 4) to ensure adequate

**Figure 1** Experimental design. n = number of rats in each group. AOM = azoxymethane.

Correspondence: R.C.N. Williamson, University Department of Surgery, Bristol Royal Infirmary, Bristol BS2 8HW, UK.
*Present address: University Department of Clinical Surgery, Royal Infirmary, Edinburgh EH3 9YW, UK.
Received 3rd June 1986; and in revised form 31st October 1986.

space and ventilation for animals within the apparatus and to avoid excessive humidity and condensation. During the first half of the experimental period thermostat malfunction led to fluctuations in temperature, and the animals were removed from the cabinet while repairs were effected during the 11th, 12th and 17th weeks (Figure 2). However, during the final 12 weeks the temperature remained within the acceptably narrow range of 7–10°C.

*Carcinogen*

At the start of the experiment rats in groups 2 and 4 received the first of 6-weekly i.p. injections of azoxymethane (total dose 90 mg $kg^{-1}$). Groups 1 (normothermic) and 3 (hypothermic) received injections of vehicle (water) and served as controls (Figure 1).

*Autopsy specimens*

All animals were killed at 30 weeks. At autopsy the entire intestinal tract was excised. The duodenum, jejunoileum, caecum and colorectum were flushed with saline to remove all content, blotted dry and weighed. The length of each segment was determined by suspension with a constant weight (9.5 g) against a ruler. The weights of the liver, kidneys and spleen were recorded. After opening the intestine longitudinally, tumours were excised and the remaining bowel was blotted dry and weighed. All tumours were fixed in 10% formalin before histological processing. Subsequently 5 μm sections were prepared for staining with haematoxylin and eosin.

In addition, 6 rats from each group received the stathmokinetic agent vincristine (1 mg $kg^{-1}$ i.p.) and were killed at half-hourly intervals from 30–180 min later. Autopsy was performed as above, but after excision of tumours the following bowel segments were fixed in Carnoy's solution: duodenum, jejunum, ileum, caecum and proximal, middle and distal thirds of the colorectum. After 3–6 h these specimens were transferred to 70% alcohol in which they could be preserved indefinitely. Later, after staining with Schiff's reagent, 10 individual crypts were isolated from the midpoint of each specimen by microdissection. The number of arrested metaphases counted in each crypt was plotted against time of death after vincristine. The crypt cell production rate (CCPR) was calculated from the slope of the least squares regression line that best fitted the data (Al-Mukhtar et al., 1982).

**Figure 2** Weekly mean temperatures in hypothermia apparatus. Dotted lines represent periods during which animals were removed while repairs were effected.

*Statistics*

Differences in crypt cell production rate were assessed using Student's *t*-test to compare regression lines. All other results were analysed using Student's *t*-test.

### Results

*Food intake and body weight*

There were no premature deaths. Hypothermic rats ate consistently more food throughout the experiment. Overall they consumed 42% more chow than those kept at normal temperature. Despite this hyperphagia, weekly increments in body weight were slightly smaller in the two hypothermic groups. By the end of the experiment these rats weighed 13% less overall (542±35 g, mean±s.e.) than normothermic animals (613±49 g, $P<0.01$). Azoxymethane had no detectable effect on body weight irrespective of ambient temperature. There were no significant differences between the four groups in weights of the liver, kidneys and spleen.

*Intestinal adaptation*

The lengths and weights of the duodenum, jejunoileum and colorectum and the weight of the caecum were unaffected by either hypothermia or azoxymethane. In the small bowel the combination of hypothermia and azoxymethane increased CCPR in the duodenum by 170% ($P<0.01$), in the jejunum by 172% ($P<0.01$), and in the ileum by 74% (Figure 3). Azoxymethane alone produced a nonsignificant (29%) increase in duodenal CCPR but had no effect in the jejunum or ileum. Hypothermia alone did not affect small bowel CCPR.

By contrast, azoxymethane increased CCPR throughout the large bowel at normal temperature (Figure 4). Increments were larger in the middle (226%; $P<0.05$) and distal colonic (156%) segments than in the caecum (84%) and proximal colon (76%). Hypothermia augmented this tropic effect by 86% in the proximal colon but not in the other three segments. In rats not receiving carcinogen colorectal CCPRs were unaffected by hypothermia.

*Carcinogenesis*

In rats receiving azoxymethane (groups 2 and 4), hypothermia increased the mean number of colorectal tumours per rat from 1.7±0.3 to 2.3±0.8 (0.6±0.2 to 0.9±0.3 in the proximal colon, but this difference did not reach statistical significance. Tumour distribution was identical, 40% of tumours being found in the proximal half of the large bowel in each group. Hypothermia did increase the diameter of colorectal tumours by 42% (3.1±0.3 to 4.4±0.5 mm) ($P<0.05$). This difference was particularly marked in the proximal colon (3.7±0.3 to 6.1±0.9 mm), where tumours were 65% larger ($P<0.05$).

No tumours were found in the duodenum or jejunoileum of azoxymethane-treated rats or at any site in rats not receiving carcinogen. Histological types of benign and malignant neoplasms were as previously described (Williamson & Rainey, 1984).

### Discussion

Although hypothermia caused marked hyperphagia in this experiment, it only increased crypt cell production rates in the presence of a further stimulus, *viz*, azoxymethane. This observation differs from previous work showing increased mucosal wet weight, villous height and crypt cell production rate in the small bowel of cold-acclimatised rats (Jacobs et al., 1982; Sagor et al., 1982; Heroux & Gridgeman, 1958; Jacobs & Dowling, 1982). In these studies, however, animals were housed at lower temperatures (5–6°C) than in the present experiment, in which technical problems prevented sustained temperatures below 10°C. This difference might reasonably explain the limited adaptive response, although increases in food intake were of the same order.

Interestingly, relative hypothermia appears to be a potent stimulus of intestinal cell proliferation in azoxymethane-treated rats. This combined effect is maximal in the upper

**Figure 3** Crypt cell production rates (CCPR) in three small bowel segments. Shaded columns represent hypothermic groups. AOM = azoxymethane. *$P < 0.01$ *vs.* controls.

**Figure 4** Crypt cell production rates (CCPR) in four large bowel segments. Shaded columns represent hypothermic groups. AOM = azoxymethane. ●$P < 0.05$ *vs.* controls.

small bowel, where CCPR is twice that of normothermic controls. It is detectable in the ileum and proximal colon as well, but absent in the remainder of the large bowel. Azoxymethane and its analogues cause acute mucosal destruction followed by compensatory hyperplasia throughout the intestinal tract (Sunter, 1980). This hyperplastic response is evident within a week of the first injection and continues up to and beyond the development of tumours (Wright, 1983). These changes tend to be maximal in segments particularly susceptible to carcinogenesis (Zedeck & Brown, 1977). We found the tropic effect of azoxymethane (alone) mainly in the large bowel and to a lesser extent in the duodenum, but absent in the jejunum and ileum, segments that are relatively resistant to chemical carcinogenesis. Nevertheless, it seems likely that azoxymethane does produce transient jejunoileal hyperplasia, which may be undetectable by 30 weeks but is enhanced and prolonged by a second stimulus such as hypothermia. By contrast, in the large bowel hypothermia augments the stimulatory effect of azoxymethane only in the proximal colon. The mucosa of the distal colon is generally less sensitive to adaptive stimuli (Bristol & Williamson, 1984), and since it responds briskly to the carcinogen alone it may be refractory to further acceleration of cell turnover by hypothermia.

Since hypothermia has a relatively modest effect on epithelial cell proliferation, it is not suprising that it does not increase the overall yield of intestinal tumours. Larger groups might have revealed more convincing evidence of the weak tumour-promoting potential reflected in the increased diameter of the proximal colonic tumours, but the size of the hypothermic cabinet restricted the accommodation of rats under humane conditions. It is notable that this co-carcinogenic effect is confined to the segment of bowel in which the separate tropic actions of azoxymethane and hypothermia overlap. These results suggest that although the tropic potential of hypothermia is limited, it is sufficient to promote earlier development or more rapid growth of tumours in the proximal colon. Intestinal adaptation stimulated by non-operative means could therefore have a similar role to that induced by surgery in promoting experimental carcinogenesis.

This study was supported by grants from the Cancer Research Campaign and the South West Regional Health Authority, UK. We thank Mrs C. Williams for technical assistance. Figures were supplied by the Departments of Medical Illustrations, Bristol Royal Infirmary, and the University of Edinburgh.

### References

AL-MUKHTAR, M.Y.T., POLAK, J.M., BLOOM, S.R. & WRIGHT, N.A. (1982). The search for appropriate measurements of proliferative and morphological status in studies in intestinal adaptation. In *Mechanisms of Intestinal Adaptation*, Robinson *et al.* (eds) p. 3. MTP Press Ltd: Lancaster.

BARTHOLD, S.W. & JONAS, A.M. (1977). Morphogenesis of early 1,2-dimethylhydrazine-induced lesions and latent period reduction of colon carcinogenesis in mice by a variant of Citrobacter freundi. *Cancer Res.* **37**, 4352.

BRISTOL, J.B. & WILLIAMSON, R.C.N. (1984). Large bowel growth. *Scand. J. Gastroenterol.* **19**, suppl. 93, 25.

ELIAS, E. & DOWLING, R.H. (1976). The mechanism for small-bowel adaptation in lactating rats. *Clin. Sci. Med.*, **51**, 427.

HEROUX, O. & GRIDGEMAN, N.T. (1958). The effect of cold acclimation and the size of organs and tissues of the rat, with special reference to modes of expression of results. *Can. J. Biochem. Physiol.*, **36**, 209.

HIROSE, F., FUKAZAWA, K., WATANABE, H., TERADA, Y., FUJII, I. & OTSUKA, S. (1977). Induction of rectal carcinoma in mice by local X-irradiation. *Gann*, **68**, 669.

JACOBS, L.R. & DOWLING, R.H. (1982). Relative roles of luminal nutrition and pacreaticobiliary secretions in regulating intestinal growth and function in the cold-acclimated rat. In *Mechanisms of Intestinal Adaptation*, Robinson *et al.* (eds) p. 433. MTP Press Ltd: Lancaster.

JACOBS, L.R., POLAK, J.M., BLOOM, S.R. & DOWLING, R.H. (1982). Intestinal mucosal and fasting plasma levels of immunoreactive enteroglucagon in three models of intestinal adaptation: resection, hypothermic hyperphagia and lactation in the rat. In *Mechanisms of Intestinal Adaptation*, Robinson *et al.*, (eds) p. 231. MTP Press Ltd: Lancaster.

MILLER, D.L., HANSON, W., SCHEDL, H.P. & OSBORNE, J.W. (1977). Proliferation rate and transit time of mucosal cells in small intestine of the diabetic rat. *Gastroenterology*, **73**, 1326.

RIJKE, R.P.C., PLAISIER, H., HOOGEVEEN, A.T., LAMERTON, L.F. & GALJAARD, H. (1975). The effect of continuous irradiation on cell proliferation and maturation in small intestinal epithelium. *Cell Tissue Kinet*, **8**, 441.

SAGOR, G.R., AL-MUKHTAR, M.Y.T., GHATEI, M.A., WRIGHT, N.A. & BLOOM, S.R. (1982). The effect of altered luminal nutrition on cellular proliferation and plasma concentrations of enteroglucagon and gastrin after small bowel resection in the rat. *Br. J. Surg.*, **69**, 14.

SHARP, J.G., CROUSE, D.A., JACKSON, J.D., MANN, S.L. & MURPHY, B.O. (1985). Abdominal irradiation is a potent promoter of dimethylhydrazine induced colon tumors in rats. *Proc. Am. Assoc. Cancer Res.*, **26**, 144.

SUNTER, J.P. (1980). Experimental carcinogenesis and cancer in the rodent gut. In *Cell Proliferation in the Gastrointestinal Tract*, Appleton *et al.* (eds) p. 256. Pitman Medical: Tunbridge Wells.

THOMPSON, M.H. (1982). The role of diet in relation to faecal bile acid concentration and large bowel cancer. In *Colonic Carcinogenesis*, Malt, R.A. & Williamson, R.C.N. (eds) p. 49. MTP Press Ltd: Lancaster.

UMPLEBY, H.C., BRISTOL, J.B., RAINEY, J.B. & WILLIAMSON, R.C.N. (1984). Survival of 727 patients with single carcinomas of the large bowel. *Dis. Colon Rectum*, **27**, 803.

WILLIAMSON, R.C.N. (1978). Intestinal adaptation. 1. Structural, functional and cytokinetic changes. *N. Engl. J. Med.*, **298**, 1393.

WILLIAMSON, R.C.N. (1982). Intestinal adaptation: Factors that influence morphology. *Scand. J. Gastroenterol*, **17**, suppl. 74, 21.

WILLIAMSON, R.C.N. & RAINEY, J.B. (1984). The relationship between intestinal hyperplasia and carcinogenesis. *Scand. J. Gastroenterol*, **19**, suppl. 104, 57.

WRIGHT, N.A. (1983). The histiogenesis of gastrointestinal cancer. In *Advances in Gastroenterology: Gastrointestinal and Hepatobiliary Cancer*, Hodgson, H.J.F. & Bloom, S.R. (eds) p. 79. Chapman & Hall: London.

ZEDECK, M.S. & BROWN, G.B. (1977). Methylation of intestinal and hepatic DNA in rats treated with methylazoxymethanol acetate. *Cancer*, **40**, 2580.